# Solution structure of the cold-shock-like protein from *Rickettsia rickettsii*

**DOI:** 10.1107/S174430911203881X

**Published:** 2012-10-26

**Authors:** Kyle P. Gerarden, Andrew M. Fuchs, Jonathan M. Koch, Melissa M. Mueller, David R. Graupner, Justin T. O’Rorke, Caleb D. Frost, Heather A. Heinen, Emily R. Lackner, Scott J. Schoeller, Paul G. House, Francis C. Peterson, Christopher T. Veldkamp

**Affiliations:** a Department of Chemistry, University of Wisconsin-Whitewater, 800 West Main Street, Whitewater, WI 53190, USA; b Department of Biological Sciences, University of Wisconsin-Whitewater, 800 West Main Street, Whitewater, WI 53190, USA; c Department of Biochemistry, The Medical College of Wisconsin, 8701 Watertown Plank Road, Milwaukee, WI 53226, USA

**Keywords:** NMR, cold-shock domains, OB folds, Rocky Mountain spotted fever, *Rickettsia rickettsii*

## Abstract

Rocky Mountain spotted fever is caused by *Rickettsia rickettsii* infection. *R. rickettsii* can be transmitted to mammals, including humans, through the bite of an infected hard-bodied tick of the family Ixodidae. Since the *R. rickettsii* genome contains only one cold-shock-like protein and given the essential nature of cold-shock proteins in other bacteria, the structure of the cold-shock-like protein from *R. rickettsii* was investigated. With the exception of a short α-helix found between β-strands 3 and 4, the solution structure of the *R. rickettsii* cold-shock-like protein has the typical Greek-key five-stranded β-barrel structure found in most cold-shock domains. Additionally, the *R. rickettsii* cold-shock-like protein, with a Δ*G* of unfolding of 18.4 kJ mol^−1^, has a similar stability when compared with other bacterial cold-shock proteins.

## Abstract 2

The solution structure of the cold-shock-like protein from *R. rickettsii*, the causative agent of Rocky Mountain spotted fever, is reported.

## 1. Introduction   

In 1906, Howard Ricketts discovered the bacterium that causes Rocky Mountain spotted fever and this bacterium was ultimately named *Rickettsia rickettsii* after him (Gross & Schäfer, 2011 ▶; Ricketts, 1906*a* ▶,*b* ▶). *R. rickettsii* is an intracellular pathogen that is transmitted to mammals, including humans, through the bite of an infected tick (Gross & Schäfer, 2011 ▶; Dumler & Walker, 2005 ▶). Even with treatment, 5–10% of humans with an *R. rickettsii* infection will die of Rocky Mountain spotted fever (Dumler & Walker, 2005 ▶). Ticks can become infected either through bacterial transfer from an infected tick to its eggs or by transfer from a tick biting an infected mammal (McDade & Newhouse, 1986 ▶; Azad & Beard, 1998 ▶; Burgdorfer & Varma, 1967 ▶). It is interesting that environmental stimuli, such as the changes in temperature that the bacterium might experience while residing in a tick or when transferred to a mammalian host, cause limited change in relative mRNA transcription levels in *R. rickettsii* (Ellison *et al.*, 2009 ▶). In particular, the relative mRNA transcription levels of the *R. rickettsii* cold-shock-like protein (*Rr*-Csp) are not significantly changed at numerous temperatures (Ellison *et al.*, 2009 ▶). It may be that, as with other cold-shock proteins, protein levels of *Rr*-Csp are determined based on translational control *versus* transcriptional control (Giuliodori *et al.*, 2004 ▶, 2010 ▶; Horn *et al.*, 2007 ▶).

Traditionally, cold-shock proteins are thought to function as RNA chaperones during cold shock through melting and binding to mRNA, thereby reducing or preventing the formation of mRNA secondary structure (Horn *et al.*, 2007 ▶; Chaikam & Karlson, 2010 ▶). This allows continued translation during cold adaptation (Horn *et al.*, 2007 ▶; Chaikam & Karlson, 2010 ▶). Cold-shock proteins have a cold-shock domain structure, also termed an oligosaccharide/oligo­nucleotide (OB) binding fold, which consist of a five-stranded antiparallel β-barrel that binds to single-stranded nucleic acids (Horn *et al.*, 2007 ▶; Chaikam & Karlson, 2010 ▶). This domain architecture is also found in eukaryotes, including plants and animals (Horn *et al.*, 2007 ▶; Chaikam & Karlson, 2010 ▶). Other conserved structural features of cold-shock proteins include two nonspecific RNA-binding sequence motifs, RNP1 and RNP2 (ribonucleoprotein motifs 1 and 2, respectively), which are involved in binding to single-stranded RNA or DNA (Horn *et al.*, 2007 ▶; Chaikam & Karlson, 2010 ▶). Given that *Rr*-Csp is the only cold-shock-like protein in the *R. rickettsii* genome (Ellison *et al.*, 2009 ▶) and given the essential nature of bacterial cold-shock proteins for bacterial survival, the solution structure of the *R. rickettsii* cold-shock-like protein and its Δ*G* of unfolding are presented and compared with those of other bacterial cold-shock proteins.

## 2. Materials and methods   

Undergraduate students taking a biochemistry laboratory course and/or a physical chemistry laboratory course completed the majority of these experiments during a single academic semester.

### 2.1. Protein expression and purification   

A codon-optimized gene coding for a SUMO-*Rr*-Csp fusion was obtained from GenScript (Piscataway, New Jersey, USA). The UniProt code for *Rr*-Csp is A8GT84_RICRS. *Escherichia coli* BL21 (DE3) cells containing the pET28a-His_6_-SUMO-*Rr*-Csp construct were grown in Luria broth or [U-^15^N/^13^C] M9 minimal medium to an OD_600_ of ∼0.6 before protein expression was induced for 4–5 h using 1 m*M* isopropyl β-d-1-thiogalactopyranoside. Cell pellets were collected by centrifugation and stored at 253 K until processing. Cells were resuspended in buffer *A* (50 m*M* sodium phosphate, 300 m*M* NaCl, 10 m*M* imidazole pH 8.0), lysed by sonication and clarified using centrifugation (15 000*g* for 15 min). The supernatant was loaded onto ∼2 ml His60 nickel resin for 30 min and the column was washed with 30 ml buffer *A*. The His_6_-SUMO-*Rr*-Csp fusion protein was eluted using a buffer consisting of 50 m*M* sodium phosphate, 300 m*M* NaCl, 250 m*M* imidazole pH 8.0. Fractions containing His_6_-­SUMO-*Rr*-Csp based on SDS–PAGE were pooled with 400 µg SUMO/ubiquitin-like protease 1 (Ulp-1) and dialyzed against 20 m*M* sodium phosphate, 50 m*M* NaCl pH 8.0 for 2 d at 277 K. After dialysis and digestion with Ulp-1, the dialysate was loaded onto a His60 nickel resin column and the flowthrough and buffer *A* wash were collected. The flowthrough and wash were exchanged into buffer consisting of 20 m*M* sodium phosphate, 50 m*M* NaCl pH 6.0 and concentrated to ∼600 µl using ultrafiltration. The molecular weight of purified *Rr*-Csp was confirmed using MALDI-TOF mass spectrometry (measured *m*/*z* 7771.1, expected *m*/*z* 7770.7). Size-exclusion chromatography was performed in 200 m*M* sodium phosphate pH 7.5 using a Zorbax GF-450 column at a flow rate of 0.5 ml min^−1^.

### 2.2. NMR spectroscopy and structure determination   

Data were acquired at the Medical College of Wisconsin’s NMR facility on a Bruker 500 MHz spectrometer equipped with a triple-resonance cryoprobe and were processed using *NMRPipe* (Delaglio *et al.*, 1995 ▶). A complete list of the collected NMR spectra can be found in the Supplementary Material^1^. The NMR sample consisted of 1.5 m*M* *Rr*-Csp in 20 m*M* sodium phosphate pH 6.0 with 50 m*M* NaCl, 10% D_2_O and 0.2% NaN_3_. All NMR spectra were collected at a sample temperature of 298 K. Backbone chemical shift assignments were generated by *GARANT* (Bartels *et al.*, 1996 ▶). Manual checking of the backbone chemical shift assignments indicated that they were correct. Side chains were assigned manually and overall assignments were 98% complete. ^1^H^α^, ^13^C^α^, ^13^C^β^, ^13^C′ and ^15^N chemical shifts and *TALOS*+ were used to generate backbone dihedral angle constraints (Shen *et al.*, 2009 ▶). Distance restraints were generated from three-dimensional ^15^N-edited NOESY–HSQC, ^13^C-edited NOESY–HSQC and ^13^C(aromatic)-edited NOESY–HSQC spectra (τ_mix_ = 80 ms). The *NOEASSIGN* module of the torsion-angle dynamics program *CYANA* 3.0 was used to assign the NOESY spectra, determine initial distance restraints and calculate initial structures (Herrmann *et al.*, 2002 ▶). *CYANA* 3.0 was used for subsequent manual refinement (Herrmann *et al.*, 2002 ▶). However, the initial ensembles calculated using the *NOEASSIGN* module had high precision and almost no constraint violations (target-function values of 0.01 or 0.00 Å^2^) and required little manual refinement. The *X-­PLOR* program was used for further refinement of the protein structure in explicit water solvent by adding physical force-field terms to the experimental constraints (Linge *et al.*, 2003 ▶; Brünger, 1992 ▶; Brunger, 2007 ▶; Schwieters *et al.*, 2003 ▶). Table 1 ▶ lists the statistics from the *PSVS* suite (Bhattacharya *et al.*, 2007 ▶), *PROCHECK-NMR* (Laskowski *et al.*, 1996 ▶) and *WHAT_CHECK* (Hooft *et al.*, 1996 ▶) for validation of the final 20 conformers, which were the 20 lowest energy conformers of the 100 calculated. Heteronuclear NOE spectra were obtained using the Bruker hsqcnoef3gpsi pulse program.

### 2.3. Δ*G* of unfolding   

A two-state equilibrium between native and denatured *Rr*-Csp was assumed, similar to other Csp studies (Motono *et al.*, 2008 ▶; Kumar *et al.*, 2001 ▶; Perl *et al.*, 2000 ▶). Guanidine-denaturation curves were used to determine the Δ*G* of unfolding of 5 µ*M* *Rr*-Csp in 100 m*M* sodium phosphate at pH 7.0 as described by Shirley (1995 ▶). Δ*G* was determined at various guanidine hydrochloride concentrations using the equation
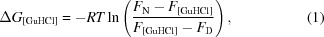
where *F*_N_ is the intrinsic tryptophan fluorescence intensity at 350 nm of the native protein, *F*_D_ is the fluorescence of the denatured protein and *F*_[GuHCl]_ is the fluorescence at a given guanidine concentration. Fluorescence intensities were measured using an F2500 Hitachi fluorescence spectrophotometer with an excitation wavelength of 285 nm and an emission scan from 300 to 500 nm. The Δ*G* of denaturation under native conditions was extrapolated using a plot of Δ*G*_[GuHCl]_ *versus* guanidine hydrochloride concentration (Shirley, 1995 ▶).

## 3. Results and discussion   

**Figure 1:**
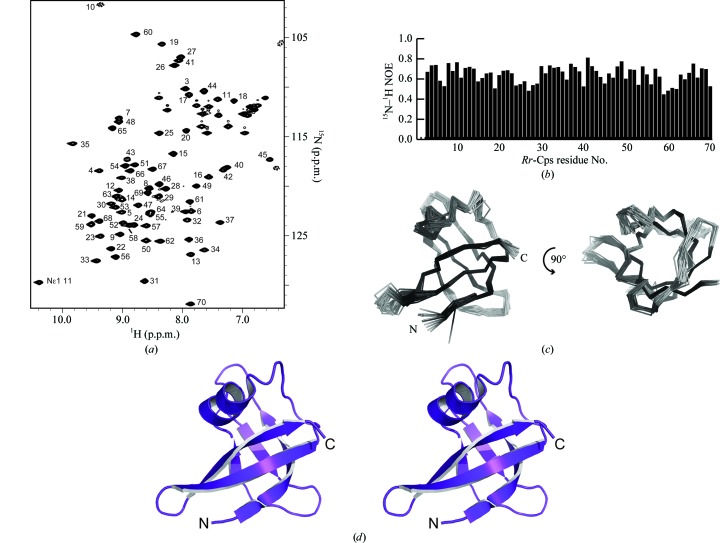
Solution structure of the *R. rickettsii* cold-shock-like protein (*Rr*-Csp). (*a*) ^15^N–^1^H HSQC spectrum acquired from 1.5 m*M* [U-^15^N/^13^C] *Rr*-Csp at 298 K using a 500 MHz Bruker spectrometer. (*b*) ^15^N–^1^H heteronuclear NOEs plotted for each *Rr*-Csp residue. (*c*) Ensemble of 20 *Rr*-Csp structures shown as two views, with the second being a 90° rotation along the *x* axis with respect to the first. (*d*) A stereoview of the lowest energy structure from the *Rr*-Csp ensemble shown as a ribbon diagram.

*Rr*-Csp, which consists of 70 residues, was purified as a His_6_-SUMO fusion using immobilized metal-affinity chromatography. Incubation with His_6_-Ulp-1 and subsequent immobilized metal-affinity chromatography was used to separate the His_6_-SUMO fusion from *Rr*-Csp (Supplementary Fig. S1 ▶ 1). After concentration and buffer exchange, the resulting two-dimensional ^15^N–^1^H HSQC spectrum of *Rr*-Csp showed a homogenous spectrum with distinct peaks distributed throughout the spectrum, indicating that the protein was folded (Fig. 1 ▶ *a*). In size-exclusion chromatography *Rr*-Csp eluted at a higher retention time than SUMO (small ubiquitin-like modifier), a monomeric protein of 11 kDa, suggesting that *Rr*-Csp is monomeric like nearly all of its bacterial homologs (Horn *et al.*, 2007 ▶). Heteronuclear NOE values indicated that the entire *Rr*-Csp protein was structured (Fig. 1 ▶ *b*). Standard NMR techniques were used to solve the structure of *Rr*-Csp (Markley *et al.*, 2003 ▶), and the *Rr*-Csp ensemble of structures was deposited in the Protein Data Bank (Bernstein *et al.*, 1977 ▶) as entry 2lss. Chemical shift assignments and structural restraints were deposited in the Biological Magnetic Resonance Bank (Ulrich *et al.*, 2008 ▶; BMRB entry 18442). Two orientations of the ensemble are shown in Fig. 1 ▶(*c*) and a stereo image of the *Rr*-Csp structure is shown in Fig. 1 ▶(*d*). As expected, *Rr*-Csp shows the conserved five-stranded β-barrel fold characteristic of cold-shock proteins, with the exception of a short α-helix between strands 3 and 4. This helix was first identified by *TALOS*+ (Shen *et al.*, 2009 ▶) and was confirmed by NOE patterns consistent with an α-helix in the ^15^N NOESY.

Although the NMR spectra indicated single peaks for each NMR-active atom, they also indicated that an unidentified molecule copurified with *Rr*-Csp. This was most apparent in the ^13^C HCCH–TOCSY, which showed signals with carbon and proton chemical shifts that most closely matched shifts common to nucleic acids. Most notable were two C atoms with chemical shifts at 89.8 p.p.m. and 87.2 p.p.m., shifts that are consistent with those observed for the C1′ and C4′ atoms of nucleotides (Supplementary Fig. S2^1^). No C atoms from proteins resonate in this region. Given that *Rr*-Csp is a cold-shock domain, also known as an oligosaccharide/oligonucleotide (OB) binding fold, it is possible that this signal comes from a nucleotide that copurified with *Rr*-Csp. Like other cold-shock proteins, *Rr*-Csp contains an RNP1-like motif (amino acids 17–23) and an RNP2-like motif (amino acids 30–34). The RNP1 and RNP2 motifs in other cold-shock proteins form a single-stranded nucleic acid-binding site (Horn *et al.*, 2007 ▶; Chaikam & Karlson, 2010 ▶; Max *et al.*, 2006 ▶). However, we were not able to identify NOEs between this copurifying molecule and *Rr*-Csp. With the exception of observing that the chemical shifts of the molecule are consistent with those of a nucleotide, identifying the molecule is beyond the scope of this structure report.

**Figure 2:**
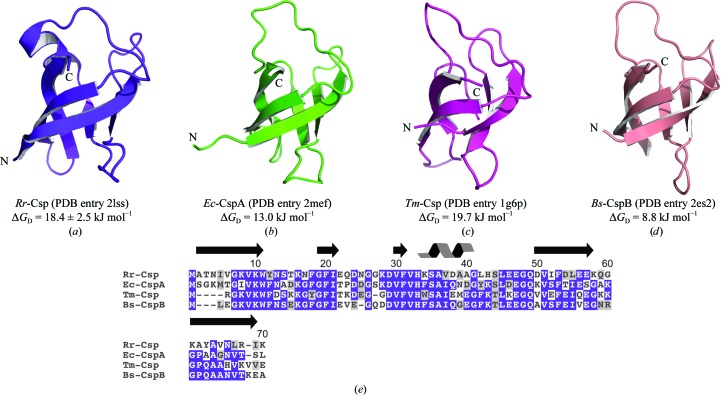
*Rr*-Csp and structural homologs with known Δ*G*_D_ values. Ribbon diagrams and Δ*G*_D_ values at room temperature for (*a*) *Rr*-Csp (PDB entry 2lss), (*b*) *E. coli* CspA (*Ec*-CspA; PDB entry 3mef; Feng *et al.*, 1998 ▶), (*c*) *T. maritima* Csp (*Tm*-Csp; PDB entry 1g6p; Kremer *et al.*, 2001 ▶) and (*d*) *B. subtilis* CspB (*Bs*-CspB; PDB entry 2es2; Max *et al.*, 2006 ▶). The Δ*G*_D_ values for *E. coli* CspA, *T. maritima* Csp and *B. subtilis* CspB are from Kumar *et al.* (2001 ▶). (*e*) Multiple sequence alignment of *Rr*-Csp with *E. coli* CspA, *T. maritima* Csp and *B. subtilis* CspB. The backgrounds of identical residues are blue and those of conserved residues are gray. The residue numbering corresponds to that of *Rr*-Csp.

In addition to being studied for their nucleic acid-binding properties and their roles in bacterial cold adaption, bacterial cold-shock proteins from mesophiles, thermophiles and hyperthermophiles have been investigated for factors that contribute to the stability and thermostability of a protein (Motono *et al.*, 2008 ▶; Kumar *et al.*, 2001 ▶; Perl *et al.*, 2000 ▶). As shown in Fig. 2 ▶, *Rr*-Csp is homologous in both sequence and structure to other bacterial cold-shock proteins from *E. coli*, *Thermotoga maritima* and *Bacillus subtilis* for which the values of Δ*G* of denaturation (Δ*G*_D_) at room temperature are known (Kumar *et al.*, 2001 ▶). To allow a comparison, the Δ*G*_D_ at room temperature for *Rr*-Csp was determined (Supplementary Fig. S3^1^; Shirley, 1995 ▶). At 18.4 ± 2.5 kJ mol^−1^, the Δ*G*_D_ value for *Rr*-Csp is higher than the values reported for other cold-shock proteins from mesophiles, such as 13.0 kJ mol^−1^ for *E. coli* CspA (*Ec*-CspA) and 8.8 kJ mol^−1^ for *B. subtilis* CspB (*Bs*-CspB) (Kumar *et al.*, 2001 ▶). The Δ*G*_D_ of *Rr*-Csp more closely matches that of 19.7 kJ mol^−1^ (Kumar *et al.*, 2001 ▶) for the Csp from the hyperthermophile *T. maritima*.

A potential hypothesis that might explain the Δ*G*_D_ of *Rr*-Csp being similar to the Δ*G*_D_ of a Csp from a hyperthermophile is that salt bridges may stabilize the *Rr*-Csp structure. Salt bridges have been suggested to contribute to the increased stability of proteins from thermophiles and hyperthermophiles (Kumar, Tsai & Nussinov, 2000 ▶; Kumar, Ma *et al.*, 2000 ▶; Kumar, Tsai, Ma *et al.*, 2000 ▶; Costantini *et al.*, 2008 ▶; Kumar *et al.*, 2001 ▶). *Tm*-Csp, the Csp from *T. maritima*, contains an arginine at position 2 that is conserved in and is known to increase the stability of Csps from thermophiles and hyperthermophiles (Perl *et al.*, 2000 ▶; Kremer *et al.*, 2001 ▶). Kremer and coworkers suggest that this arginine is a part of an ion cluster, a cluster of acidic and basic amino acids, that contributes to the increased stability of *Tm*-Csp (Kremer *et al.*, 2001 ▶). Two salt bridges identified in the lowest energy conformer of the solution structure of *Tm*-Csp support this claim. *Rr*-­Csp does not contain the conserved arginine found in the Csps of thermophiles and hyperthermophiles (Fig. 2 ▶ *e*). However, each structure in the *Rr*-Csp ensemble contains an average of four salt bridges. Analysis of the structures of *Bs*-CspB and *Ec*-CspA, which are Csps from mesophiles, revealed one salt bridge in *Bs*-CspB and none in *Ec*-CspA. Salt bridges were identified by the program *ESBRI* (Costantini *et al.*, 2008 ▶) using a cutoff distance of 4 Å between charged residues. One salt bridge in *Rr*-Csp was also suggested by the seven close contacts that were identified during validation of the *Rr*-­Csp ensemble. Each close contact is in a different structure of the ensemble and consists of either an H^ζ1^ or an H^ζ3^ atom of Lys10 at a distance of 1.55–1.60 Å from either the O^∊1^ or the O^∊2^ atom of Glu22. This potential salt bridge is not likely to be present in *Ec*-CspA, which contains an identical lysine but has a threonine instead of the glutamate found in *Rr*-Csp. *Bs*-CspB does contain the corresponding lysine and glutamate. However, the side-chain amino group of the lysine and the carboxyl group of the glutamate in *Bs*-CspB are 5.4 Å apart and were not identified as a salt bridge by *ESBRI* (Costantini *et al.*, 2008 ▶).

Future experiments are needed to test the hypothesis that potential salt bridges in *Rr*-Csp are responsible for the increased stability. However, this structure, which is the first solution structure from the *R. rickettsii* genome to be deposited in the PDB, provides an example of how structural biology techniques can be successfully learned and applied in an undergraduate laboratory course.

## Supplementary Material

PDB reference: cold-shock-like protein, 2lss

Supporting information file. DOI: 10.1107/S174430911203881X/kw5052sup1.pdf

## Figures and Tables

**Table 1:** Statistics for 20 *Rr*-Csp conformers (PDB entry 2lss; BMRB entry 18442)

Completeness of resonance assignments† (%)	98
Constraints	
Nonredundant distance constraints	
Total	1813
Intraresidue (*i* = *j*)	1123
Sequential [(*i* *j*) = 1]	298
Medium [1 (*i* *j*) 5]	86
Long	306
Dihedral angle constraints ( and )	120
Constraints per residue	
Average No. of constraints per residue	27
Constraint violations	
Average No. of distance-constraint violations per structure	
0.10.2	17.05
0.20.5	1.45
>0.5	0
Average r.m.s. distance violation per constraint ()	0.02
Maximum distance violation ()	0.36
Average No. of dihedral angle violations per structure	
110	3.75
>10	0
R.m.s. dihedral angle violation per constraint ()	0.33
Maximum dihedral angle violation ()	3.4
Average atomic r.m.s.d. to the mean structure ()	
Residues 270	
Backbone (C, C, N)	0.49 0.09
Heavy atoms	0.93 0.09
Deviations from idealized covalent geometry‡	
Bond-length r.m.s.d. ()	0.017
Torsion-angle violations r.m.s.d. ()	1.3
LennardJones energy§ (kJmol^1^)	1450 40
Ramachandran statistics¶ (% of all residues)	
Most favored	86.2
Additionally allowed	13.8
Generously allowed	0
Disallowed	0

† The missing chemical shifts are the H, H, Q, Q and Q of Met1, the H of Ala2 and the H of Phe31.

‡ Final *X-PLOR* (Brnger, 1992 ▶) force constants (kcalmol^1^; 1cal = 4.186J) were 250 (bonds), 250 (angles), 300 (impropers), 100 (chirality), 100 (), 50 (NOE constraints) and 200 (torsion-angle constraints). Idealized covalent geometry is from Engh Huber (1991 ▶).

§ Nonbonded energy was calculated in *X-PLOR-NIH* (Schwieters *et al.*, 2003 ▶).

¶ Values are from *PROCHECK-NMR* (Laskowski *et al.*, 1996 ▶).

## Footnotes

1 Supplementary material has been deposited in the IUCr electronic archive (Reference: KW5052).
