# Supplementary material for: Solution structure of the cold-shock-like protein from Rickettsia rickettsii
Source: Acta Crystallogr Sect F Struct Biol Cryst Commun. 2012 Oct 26;68(Pt 11):1284–8. doi: 10.1107/S174430911203881X (PMC3515365; doi:10.1107/S174430911203881X)
Supplement: Supplementary file 1 [file f-68-01284-sup1.pdf]

Supplemental Information

Solution Structure of the Cold Shock-Like Protein from *Rickettsia rickettsii*

Kyle P. Gerarden[a#], Andrew M. Fuchs[b#], Jonathan M. Koch[a#], Melissa M. Mueller[a#], David R. Graupner[a], Justin T. O'Rorke[a], Caleb D. Frost[a], Heather A. Heinen[a], Emily R. Lackner[a], Scott J. Schoeller[a], Paul G. House[a], Francis C. Peterson[c], and Christopher T. Veldkamp[a,c*]

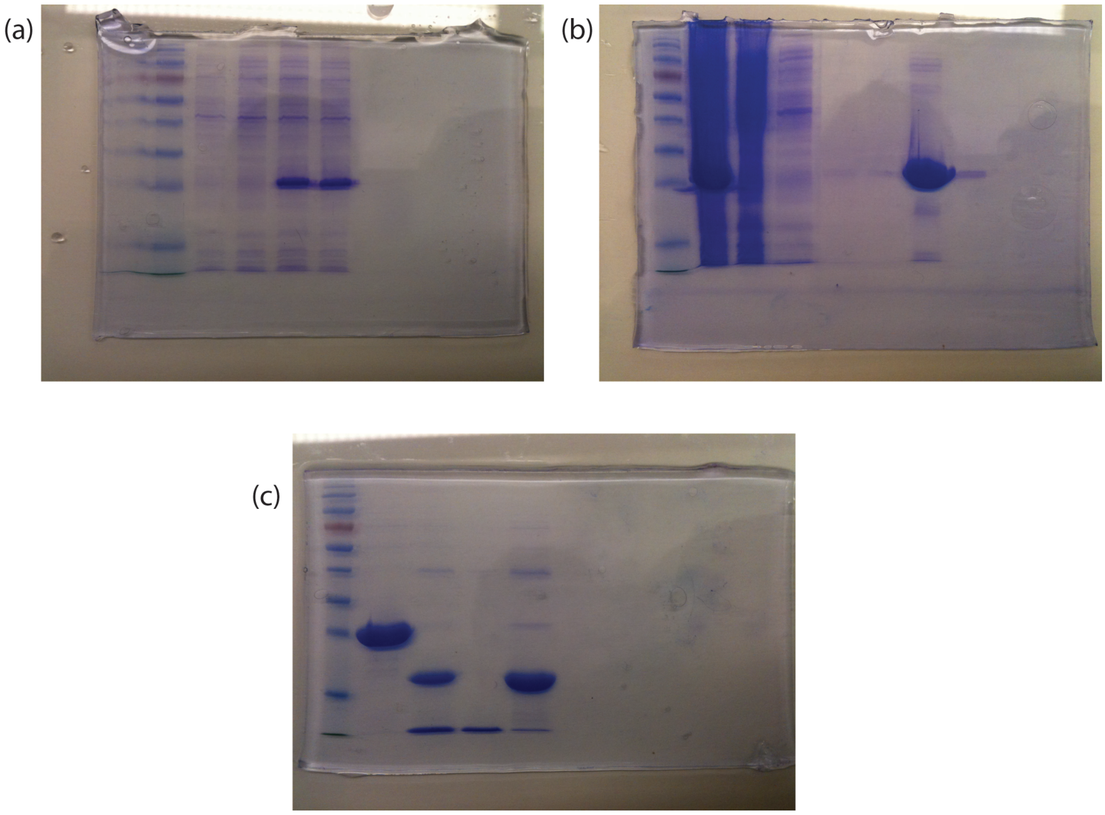

Supplemental Figure 1. Expression and Purification of *Rr*-Csp. (a) Expression of pET28a-SUMO-*Rr*-Csp. Lane 1: Ladder (11, 17, 26, 34, 43, 56, 72, 95, 130, 170 kDa), Lane 2: Un-induced total protein, Lane 3: Un-induced soluble protein, Lane 4: Total protein four hours post induction, Lane 5: Soluble protein four hours post induction. (b) Fractions from the purification of His_6-SUMO-*Rr*-Csp. Lane 1: Ladder (11, 17, 26, 34, 43, 56, 72, 95, 130, 170 kDa), Lane 2: Column load, Lane 3: Flow through, Lane 4: Wash one, Land 5: Wash two, Lane 6: Wash three, Lane 7: Elution one, Lane 8: Elution two, Lane 9: Elution three. (c) Ulp-1 digestion and reverse immobilized metal affinity purification of *Rr*-Csp. Lane 1: Ladder (11, 17, 26, 34, 43, 56, 72, 95, 130, 170 kDa), Lane 2: Undigested His_6-SUMO-*Rr*-Csp, Lane 3: Ulp-1 digested His_6-SUMO-*Rr*-Csp and column load, Lane 4: Flow through and wash (*Rr*-Csp), Land 5: Elution (His_6-SUMO).

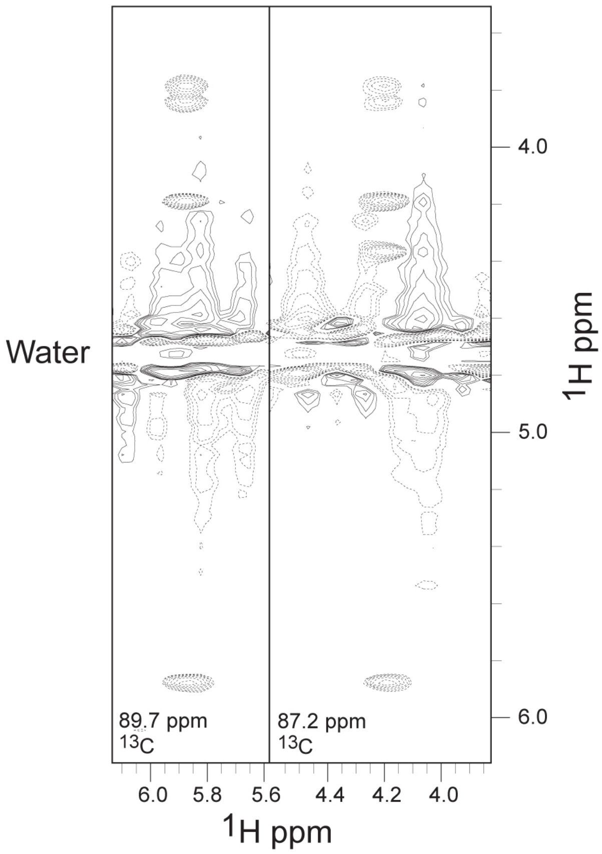

Supplemental Figure 2. Non-protein signal was observed in a 3D ^{13}C HCCH-TOCSY. Two different carbon chemical shifts at 89.7 ppm and 87.2 ppm, a region where no carbons from proteins resonate, most closely corresponds to where C1' or C4' shifts for nucleotides are observed. The proton chemical shifts also fall within expected value ranges for protons in the ribose or deoxyribose of nucleotides.

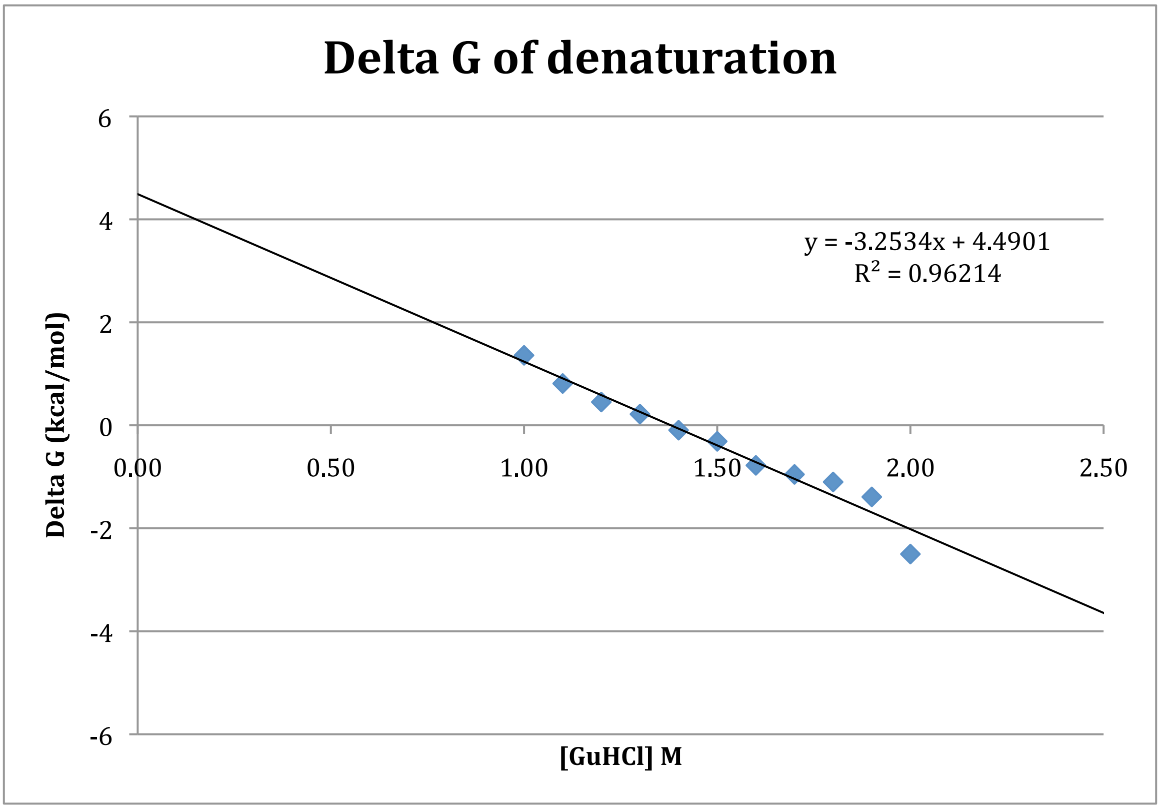

Supplemental Figure 3. A representative plot of the ΔG_D at different concentrations of guanidine HCl versus guanidine HCl concentration. A trend line was fit to the data and the equation describing the trend line was used to extrapolate the ΔG_D for *Rr*-Csp at native (0 M guanidine HCl) conditions. This plot is representative of four experiments. The reported ΔG_D is the average value for the four experiments.

List of collected NMR spectra and references

^{15}N-^{1}H HSQC, ^{13}C-^{1}H aromatic HSQC, HNCO, HN(CO)CA, HN(CO)CACB, HNCA, HNCACB, HN(CA)CO, H(CCO)NH, HBHA(CO)NH, HCCH-TOCSY, 3D ^{15}N-edited NOESY-HSQC, ^{13}C-edited NOESY-HSQC, and ^{13}C(aromatic)-edited NOESY-HSQC

Carlomagno T., Maurer M., Sattler M., Schwendinger M.G., Glaser S.J. & Griesinger C. (1996). *J. Biomol. NMR* **8**, 161-170.

Clowes R.T., Boucher W., Hardman C.H., Domaille P.J. & Laue E.D. (1993). *J Biomol NMR* **3**, 349-354.

Clubb R.T., Thanabal V. & Wagner G. (1992). *J. Magn. Reson.* **97**, 213-217.

Grzesiek S., Anglister J. & Bax A. (1993 *J. Magn. Reson. Ser. B* **101**, 114-9.

Grzesiek, S. & Bax, A. (1992). *J. Magn. Reson.* **96**, 432-440.

Grzesiek, S. & Bax, A. (1993). *J. Biomol. NMR* **3**, 185-204.

Kay, L. E., Xu, G.-Y., Singer, A. U., Muhandiram, D. R. & Forman-Kay, J. D. (1993). *J. Magn. Reson. Ser. B* **101**, 333-337.

Kay, L. E., Xu, G. Y. & Yamazaki, T. (1994). *J. Magn. Reson. Ser. A* **109**, 129-133.

Logan, T. M., Olejniczak, E. T., Xu, R. X. & Fesik, S. W. (1993). *J Biomol NMR* **3**, 225-231.

Lyons B.A. & Montelione G.T. (1993). *J. Magn. Reson. Ser. B* **101**, 206-9.

Montelione G.T., Lyons B.A., Emerson S.D. & Tashiro M. (1992). *J. Am. Chem. Soc.* **114**, 10974-75.

Muhandiram D.R., Farrow N.A., XU G.Y., Smallcombe S.H. & Kay L.E. (1993). *J. Magn. Reson. Ser. B* **102**, 317-321.

Muhandiram, D. R. & Kay, L. E. (1994). *J. Magn. Reson. Ser. B* **103**, 203-216.

Piotto, M., Saudek, V. & Sklenar, V. (1992). *J. Biomol. NMR* **2**, 661-665.

Schleucher, J. r., Sattler, M. & Griesinger, C. (1993). *Angewandte Chemie International Edition in English* **32**, 1489-1491.

Sklenar, V., Piotto, M., Leppik, R. & Saudek, V. (1993). *J. Magn. Reson.* **102**, 241-245.

Talluri, S. & Wagner, G. (1996). *J. Magn. Reson. B* **112**, 200-205.

Wittekind, M. & Mueller, L. (1993). *J. Magn. Reson. Ser. B* **101**, 201-205.

Yamazaki T., Lee W., Arrowsmith C.H., Muhandiram D.R., & Kay L.E. (1994). *J. Am. Chem. Soc.* **116**, 11655-11666.
